# Management of intrauterine adhesions using human amniotic mesenchymal stromal cells to promote endometrial regeneration and repair through Notch signalling

**DOI:** 10.1111/jcmm.17023

**Published:** 2021-11-01

**Authors:** Jie Yu, Wenwen Zhang, Jiayue Huang, Yating Gou, Congcong Sun, Yingfeng Zhang, Yanhua Mao, Benyuan Wu, Changjiang Li, Nizhou Liu, Tingting Wang, Jiren Huang, Jia Wang

**Affiliations:** ^1^ Department of Obstetrics and Gynecology University‐Town Hospital of Chongqing Medical University Chongqing China

**Keywords:** endometrial epithelial cells, hAMSCs, induction and differentiation, intrauterine adhesions, Notch signalling pathway

## Abstract

Intrauterine adhesions (IUAs) severely hamper women's reproductive functions. Human amniotic mesenchymal stromal cell (hAMSC) transplantation is effective in treating IUAs. Here, we examined the function of Notch signalling in IUA treatment with hAMSC transplantation. Forty‐five Sprague‐Dawley female rats were randomly divided into the sham operation, IUA, IUA + E2, IUA + hAMSCs and IUA + hAMSCs + E2 groups. After IUA induction in the rats, hAMSCs promoted endometrial regeneration and repair via differentiation into endometrial epithelial cells. In all groups, the expression of key proteins in Notch signalling was detected in the uterus by immunohistochemistry. The results indicated Notch signalling activation in the hAMSCs and hAMSCs + E2 groups. We could also induce hAMSC differentiation to generate endometrial epithelial cells in vitro. Furthermore, the inhibition of Notch signalling using the AdR‐dnNotch1 vector suppressed hAMSC differentiation (assessed by epithelial and mesenchymal marker levels), whereas its activation using the AdR‐Jagged1 vector increased differentiation. The above findings indicate Notch signalling mediates the differentiation of hAMSCs into endometrial epithelial cells, thus promoting endometrial regeneration and repair; Notch signalling could have an important function in IUA treatment.

## 1 INTRODUCTION

Intrauterine adhesions (IUAs), resulting from damaged basal layer of the endometrium, partially or completely obliterate the uterus and/or the cervix,^1^, ^2^ and cause infertility, oligomenorrhoea and recurrent abortions.^3^, ^4^, ^5^, ^6^ The aim of IUA treatment is to re‐establish a healthy anatomy (by adhesion removal) and reinstate uterine function.^7^, ^8^ However, prognosis following surgical restoration of the uterine cavity is poor, with up to 62.5% cases recurring.^9^ This can be attributed to a suboptimal regeneration of functional endometrum.^10^ The transplantation of mesenchymal stem cells (MSCs) into the uterine cavity could efficiently induce endometrial regeneration and remove IUAs.^11^, ^12^

Human amniotic mesenchymal stromal cells (hAMSCs) represent an ethically nonproblematic, easily isolable, abundant and immune‐privileged cell source.^13^ In addition, similar to other mesenchymal stromal cells, hAMSCs can differentiate into cells of all three germ layers in animal and cell culture experiments, exhibiting immunomodulatory properties through paracrine effects.^14^ Interestingly, hAMSCs can also be used to treat other female pathologies, including primary ovarian insufficiency and polycystic ovarian syndrome.^15^ Consequently, hAMSCs are considered useful candidates for transplantation treatment approaches and might represent a superior option compared with classical stem cells for xenografts and allografts.^16^, ^17^ Previously, we demonstrated hAMSCs can differentiate into endometrial epithelial cells, with hAMSC transplantation potentially clearing uterine adhesions.^18^

Notch receptor genes including *NOTCH1*, *NOTCH2* and *NOTCH3*, the NOTCH ligand, *JAG1* and downstream effectors *HEY1*, *HEY2* and *HEYL* are reportedly upregulated in endometrial MSCs (eMSCs). This strongly indicates that the Notch system is activated in eMSCs.^19^, ^20^ Notch1 and Jagged1 are also regulated in the endometria with UATs. A study has examined how Notch signalling regulates hAMSCs in the treatment of IUAs.^21^

There are four Notch receptors (Notch1 to Notch4) and five Notch ligands (Jagged1 and Jagged2 and DLL1, DLL3 and DLL4) in vertebrates that encompass intracellular, extracellular and transmembrane domains, all of which control multiple physiological events, including cell division and differentiation.^22^, ^23^ After binding of the Notch ligand to its receptor in neighbouring cells, the receptor undergoes cleavage at two sites, thereby releasing the Notch intracellular domain (NICD). The NICD exerts its biological effects by entering the nucleus and binding to the DNA‐binding protein CSL (RBPjk/CBF1) and the transcriptional coactivator MAM to regulate the target genes *Hes1*, *Hes5* and *Hey1*, as shown in the figure below.^24^, ^25^

In the present study, we aimed to explore the function of Notch signalling and its molecular mechanisms in hAMSC differentiation into endometrial epithelial cells and to evaluate its significance in hAMSC transplantation for IUA treatment by overexpressing and competitively inhibiting Jagged1 (ligand) and Notch1 (receptor), respectively.

## 2 MATERIALS AND METHODS

### 2.1 Animals

Forty‐five Sprague‐Dawley (SD) rats (8 weeks old, 200–220 g) were provided by the Animal Experimental Centre of Chongqing Medical University. Five rats were housed in one cage; they were maintained in a controlled environment under a 12‐h light and dark cycle at 22°C, with food and water provided *ad libitum*. The study protocol was approved by the Ethics Committee of Chongqing Medical University (20141230).

### 2.2 Isolation, culture, and identification of hAMSCs

Amniotic membrane samples were provided by the University‐town Hospital of Chongqing Medical University and the First Affiliated Hospital of Chongqing Medical University. Previously used methods for the isolation and culture of hAMSCs by our research group were applied in this study.^26^ We isolated hAMSCs from amniotic membrane specimens obtained from healthy pregnant women during full‐term caesarean section, after obtaining informed consent. Fragments (3 mm^2^) of the amniotic membranes were digested with 0.25% trypsin (BeyotimeBio, Shanghai, China) at 37°C for 30 min, followed by incubation with 0.1% type I collagenase (Meilun, Shanghai, China) at 37°C for 1 h. The obtained hAMSCs were cultured in DMEM/F12 medium (Gibco) with 10% foetal bovine serum (Gibco). Half of the medium was changed after 24 h, and the entire medium was changed after 72 h. hAMSCs were passaged when they reached >80% confluency and were selected for identification in the third passage. The expression of the markers CD29, CD44, CD73, CD105, CD19, CD34 and CD45 on the surface of hAMSCs was detected by immunofluorescence, and the differentiation characteristics of hAMSCs were examined by staining with Oil Red O and Alizarin Red (Solarbio, Shanghai, China).

### 2.3 PKH26 labelling of hAMSCs

Third‐passage hAMSCs were prepared as a single‐cell suspension, which was washed with phosphate‐buffered saline (PBS) (Solarbio, Shanghai, China) and centrifuged to obtain a cell pellet. For PKH26 labelling (Sigma), the cells were resuspended in 1 ml of Diluent C as directed by the manufacturer. Next, the labelling solution was mixed with the single‐cell suspension and incubated for 5 min. 10% foetal bovine serum (Gibco) was then added to terminate the reaction. The sample was washed thrice with 10 ml of complete medium. The labelled hAMSCs were then cultured until they became adherent. Thereafter, the nuclei were stained with 4′,6‐diamidino‐2‐phenylindole (DAPI) (Boster Biological, Shanghai, China). An observation under a fluorescent microscope (Nikon, Japan) revealed the cells were well‐labelled and adhered to the walls. The obtained hAMSCs were finally stored in an incubator until transplantation into rat uteruses.

### 2.4 Preparation of endometrium‐conditioned medium

The bilateral uteruses of 12‐week‐old female SD rats were removed under aseptic conditions after general anaesthesia with 10 ml/kg of 5% chloral hydrate (Biosharp, Shanghai, China). The uteruses were then immediately placed on ice and dissected longitudinally, cut transversely at approximately 0.3 cm in length and weighed. Next, 100 g of the prepared uterine segments was added to 1 L of ice‐cold DMEM/F12 medium; the samples were sealed and mixed on a shaker at 4°C for 2 h. After overnight incubation at 4°C, the samples were centrifuged at 18,000 ***g*** for 30 min at 4°C. Finally, the solution was filtered with a sterile filter (0.22 μm filter; Millipore) and the filtrate was stored at −20°C.^27^

### 2.5 Generation of model IUA rats

To generate IUA models, the endometria of the rats were injured with 95% alcohol. Dioestrus rats were selected based on the vaginal smear analysis and were fully anaesthetized with 10 ml/kg of 5% chloral hydrate. The skin of the lower abdomen was then exposed and disinfected with iodophor, and a longitudinal incision of 2 cm was made at the site (3 cm above the vaginal orifice). Next, the abdomen was cut open layer‐by‐layer until the uterus was exposed. The entire exposed uterus was ligated by suturing the avascular area of the mesometrium using a small round needle (with a silk thread). Next, 95% ethyl alcohol was administered by injection into the uterus with a 1‐ml empty syringe; an investigator performed clamping of the distal end of the right uterus using a pair of flat forceps. The injection was stopped after complete filling of the uterus. Uterus colour was subsequently assessed. When the uterine cavity turned white after approximately 3 min (Figure 2C), ethanol was removed followed by saline washing. After these procedures, the uterus was unclamped, unstitched and placed back into the abdomen; thereafter, the muscle and skin were sutured. The abdomen was incised again after 2 weeks to observe the morphological changes on the modelled side (Figure 2D).

### 2.6 Experimental design and treatment protocol

Forty‐five female SD rats were randomized into the sham operation, IUA, IUA + E2, IUA + hAMSCs and IUA + hAMSCs + E2 groups. Rats in the sham operation group were treated with the same procedures as the IUA models, without 95% alcohol injection. The nine rats of the IUA group received no other intervention or treatment after modelling. Two weeks after modelling, the IUA + E2 group was administered powdered oestrogen tablets (University‐town Hospital of Chongqing Medical University) mixed with water by gavage at a dose of 0.1 mg/kg once a day. Additionally, 2 weeks after the IUA modelling, the lower abdomen of rats in the IUA + hAMSCs group was cut open at the previous incision site to remove the right uterus for the injection of PKH26‐labelled hAMSCs using a 1‐ml sterile syringe at the smaller and stiffer site of the uterus (1 × 10^7^ hAMSCs were resuspended in 200 μl of PBS buffer). The uterus became swollen with the injection of hAMSCs (Figure 2E). The abdomen was stitched again after the transplantation. The rats in the IUA + hAMSCs + E2 group also underwent the same procedures of PKH26‐labelled hAMSC injection and oestrogen administration 2 weeks after the severe IUA modelling.

### 2.7 Sampling

All rats were euthanized after 4 weeks to harvest the bilateral uteruses. The uteruses of three rats randomly chosen from the IUA and IUA + hAMSCs groups, respectively, were used to prepare frozen sections. The remaining uteruses were used for paraffin‐embedded sections. The samples were stained with haematoxylin and eosin (H&E) and Masson's trichrome reagent, respectively. The distribution of PKH26‐labelled hAMSCs in the rat endometrium was observed by immunofluorescence, and the level of E‐cadherin and key Notch pathway effectors (Notch1‐4, Jagged1 and Jagged2, Hes1 and NICD) was detected by immunohistochemistry.

### 2.8 Immunofluorescence

The rat uteruses were harvested and used to prepare frozen sections 2 weeks after hAMSC transplantation. The nuclei were stained with DAPI and a fluorescent microscope was utilized to observe PKH26‐labelled hAMSCs in the uterus specimens. Frozen sections of the uteruses from the IUA models not injected with PKH26‐labelled hAMSCs were used as negative controls.

### 2.9 Histochemical staining and image analysis

The harvested uteruses were fixed with 4% formalin (Biosharp, Shanghai, China), paraffin‐embedded and sectioned. H&E staining and Masson staining were carried out with H&E and Masson staining kits, respectively (Solarbio, Shanghai, China), as directed by the manufacturer. H&E staining was applied to evaluate the number of glands in the endometrium and measure endometrial thickness; Masson staining was carried out to calculate the rate of fibrotic area (total fibrotic area of the endometrial stroma by summing the stromal and glandular endometrial areas). Microscopically, collagen fibres were blue. E‐Cadherin, Notch1, Notch2, Notch3, Notch4, Jagged1, Jagged2, NICD and Hes1 levels in the endometrium were detected using immunohistochemical kits (SP9001; ZSGB‐Bio, Shanghai, China). The main procedures performed were as follows. The sections were dewaxed, rehydrated and washed with PBS thrice for 5 min each. The antigens in the sections were recovered using EDTA antigen retrieval solution (BOSTER, Shanghai, China), followed by three PBS washes for 5 min each. Thereafter, endogenous peroxidase in the tissues was removed, with subsequent PBS washes as mentioned above. Next, blocking was performed with goat anti‐rabbit IgG, followed by incubation with primary antibodies, including rabbit anti‐E‐cadherin (1:200; CST), anti‐Notch1 (1:400; CST), anti‐Notch2 (1:250; CST), anti‐Notch3 (1:300; Abcam), anti‐Notch4 (1:100; Abcam), anti‐Jagged1 (1:200; CST), anti‐Jagged2 (1:50; CST), anti‐NICD (1:100; Abcam) and anti‐Hes1 (1:1000; Abcam) primary antibodies, respectively, incubated at 4°C overnight, followed by three PBS washes of 5 min each. The samples were subjected to subsequent incubation with biotin‐labelled goat anti‐rabbit IgG secondary antibodies at 37°C for 60 min and washed with PBS thrice (5 min each). Next, horseradish peroxidase (HRP)‐labelled streptavidin was added to the sample, which was allowed to react for 15 min; then, the samples were subjected to diaminobenzidine (ZSGB‐Bio, Shanghai, China) staining and microscopical assessment. After rinsing with ultrapure water, haematoxylin counterstaining was carried out for 20 s, which was followed by washing and microscopical assessment. Finally, routine dehydration, clearing (for transparency) and mounting were performed. For analysis, three random high‐power fields (40×) were examined per specimen. Image‐Pro Plus v6.0 was used to estimate the percentage of the positive staining area, whose average value was taken as the relative protein expression.

### 2.10 In vitro‐induced differentiation of hAMSCs

Third‐passage hAMSCs were subjected to routine culture in six‐well plates (Corning). At 30%–40% confluency, the induction group medium was changed to DMEM/F12 + 10 ng/ml TGF‐β1 + 10 ng/ml LEGF + 10 ng/ml PDGF‐BB + 1 × 10^−6^ M β‐estradiol (all from Solarbio, China) + endometrium‐conditioned medium containing 2% FBS. Control hAMSCs were incubated in DMEM/F12 alone containing 2% FBS. The medium was changed at 2 days, with the entire culture process lasting 3 days.

### 2.11 Quantitative reverse transcription‐PCR (qRT‐PCR)

The total RNA was obtained from the induction and control group cells grown in six‐well plates using the TRIzol method (Sangon Biotech, Shanghai, China) and reverse transcribed using the TAKARA reverse transcription kit (Sangon Biotech) as directed by the manufacturer. Specific primers for cytokeratin (*CK)*‐*7*, *CK*‐*8*, *CK*‐*18*, *CK*‐*19*, *E*‐*cadherin*, *Notch1* and *Jagged1* shown in Table 1 were used for PCR amplification. The PCR system included 20 μl of reagents in total, and amplification was performed at 95°C (30 s), followed by 40 cycles at 95°C (5 s) and 60°C (30 s), and final extension at 65°C. The target gene expression was determined based on the Ct values, with *GADPH* as an internal reference. Three technical replicates were used to measure the mRNA levels of *CK*‐*7*, *CK*‐*8*, *CK*‐*18*, *CK*‐*19*, *E*‐*cadherin*, *Notch1* and *Jagged1*, and this procedure was repeated three times. The relative expression was calculated using the 2^−△△Ct^ method.

**TABLE 1:** Primers utilized in quantitative real‐time PCR

	Gene	Sense (5′−3′)	Non‐sense (5′−3′)
Human	Ck‐7	TGGATGCCCTGAATGATGAGAT	GGGAGCGACTGTTGTCCA
	Ck‐8	CCATTAAGGATGCCAACGCCAA	TTCATCAGCTCCTGGTACTCAC
	Ck‐18	ATGGGAGGCATCCAGAACGA	TCTCCAAGTGCTCCCGGATT
	Ck‐19	ACTACACGACCATCCAGGAC	GCAGAGCCTGTTCCGTCTCA
	E‐Cadherin	ACCATTCAGTACAACGACCCAA	GCCTTCCTACAGACGCCAG
	Notch1	CATCACCTGCCTGTTAGGAG	ACACATGGCAACATCTAACCC
	Jagged1	GCCGTTGCTGACTTAGAATCCC	CTCGATTTCCCAGCCAACCAC
	GAPDH	AACAGCCTCAAGATCATCAGC	ATGAGTCCTTCCACGATACCAA

### 2.12 Immunofluorescence

The medium in 24‐well plates was discarded, and the remaining cells were subjected to three PBS washes for 5 min each and fixed with 4% formalin for 15 min. After a 15‐min incubation with 0.1% Triton X‐100 (V900502; Sigma), the cells were subsequently blocked with 10% goat serum (AR004) at 37°C for 30 min. Thereafter, the sections were overnight incubated with rabbit anti‐E‐cadherin (1:100; CST) and anti‐vimentin (1:100; CST) primary antibodies at 4°C, whereas PBS was added to cells in the control group. This was followed by incubation with Alexa Fluor‐linked goat anti‐rabbit IgG (1:1000, Abcam, UK) at 37°C in the dark for 2 h and four PBS washes of 5 min each. DAPI counterstaining for 10 min was performed in the dark. Finally, mounting was carried out with 20 μl of anti‐fluorescent quencher (Meilun‐Bio, Shanghai, China), and a fluorescent microscope was utilized for analysis.

### 2.13 Transfection of hAMSCs with recombinant adenovirus vectors AdR‐dnNotch1, Adr‐Jagged1 and Ad‐RFP

The recombinant adenoviral vectors AdR‐dnNotch1, Adr‐Jagged1 and Ad‐RFP were provided by Denovo (Shanghai, China). Ad‐RFP was utilized as the control virus. To assess the association of Notch signalling with hAMSC differentiation into endometrial epithelial cells, hAMSCs were assigned to the control, induction, inhibition and activation groups. The cells used were from the third passage after they adhered to the plates. The control and induction groups were transfected with Ad‐RFP. Additionally, in the induction group, medium replacement with the induction culture medium (DMEM/F12 + 10 ng/ml TGF‐β1 + 10 ng/ml LEGF + 10 ng/ml PDGF‐BB + 1 × 10^−6^ mol/L β‐estradiol + endometrium‐conditioned medium containing 2% FBS) was performed along with Ad‐RFP infection. In the inhibition group, the medium was replaced with the induction culture medium, and the cells were infected with Adr‐dnNotch1. The medium was also changed to the induction culture medium in the activation group, and the cells were infected with Adr‐Jagged1. Polymix (Denovo, Shanghai, China) was added at a 1:1 ratio to each group. Red fluorescence was observed under a fluorescence microscope 24 h after infection. More than 80% of cells were successfully infected with the viruses after 72 h, with weak fluorescence. Medium change occurred after 2 days, and cells were cultured for 3 days. The total RNA was collected for qRT‐PCR (repeated three times). The expression of vimentin and E‐cadherin proteins was detected by immunofluorescence.

### 2.14 Flow cytometry

The treated cells were incubated with 0.25% trypsin (Beyotime, Shanghai, China). The resulting cell suspension was centrifuged in pre‐cooled PBS at 200 g for 5 min for washing. A total of three washes were performed. Finally, 1 × 10^6^ cells were resuspended in 0.1 ml of PBS, and 500 μl chilled 75% ethanol (Chongqing Chuandong Chemical Co., Ltd.) was added dropwise to prevent cell aggregation. The percentage of cells in various phases (G0/G1, S and G2/M) was analysed by flow cytometry in triplicate assays.

### 2.15 Statistical analysis

Data were analysed using SPSS 21.0. Normally distributed measurement data are presented as mean (x) ± standard deviation (sd). The one‐way ANOVA and *t* test were carried out to compare multiple groups and group pairs, respectively. Histograms were drawn with Image‐Pro Plus 6.0. *p* < 0.05 indicated statistical significance.

## 3 RESULTS

### 3.1 Identification of hAMSCs

**FIGURE 1:**
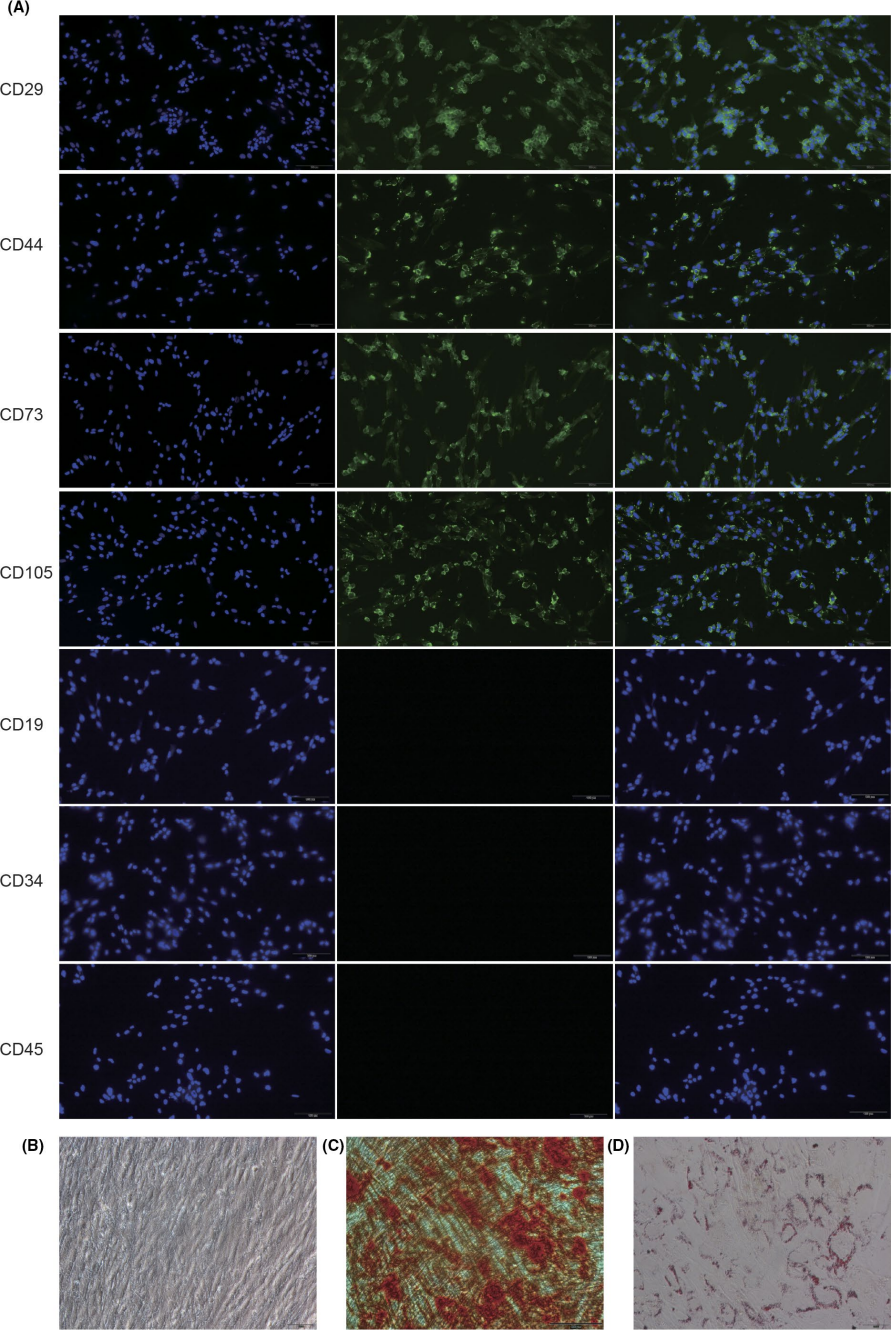
Morphology and identification of hAMSCs. (A) Detection of CD29, CD44, CD73, CD105, CD19, CD34 and CD45 in hAMSCs by immunofluorescence. (B) The third‐passage hAMSCs were fusiform fibroblasts. (C) Results of alizarin red staining of hAMSCs after being cultured in osteogenic medium for 21 days. (D) Results of Oil Red O staining of hAMSCs after being cultured in adipocyte medium for 14 days

The morphology of most adherent hAMSCs was similar to that of fibroblasts (Figure 1B). Immunofluorescence revealed that hAMSCs expressed the stemness biomarkers CD29, CD44, CD73 and CD105, but did not express CD19, CD34 and CD45 (Figure 1A). To confirm that hAMSCs have differentiation ability, we induced hAMSC differentiation to osteoblasts and adipocytes. hAMSCs in the osteogenic differentiation medium were stained with Alizarin Red after culturing for 21 days, and orange‐red nodules were observed (Figure 1C). hAMSCs in the adipocyte differentiation medium were stained with Oil Red O after being cultured for 14 days, and red fat drops were observed (Figure 1D).

### 3.2 Promotion of endometrial epithelial cell regeneration and repair by in vivo transplantation of hAMSCs

**FIGURE 2:**
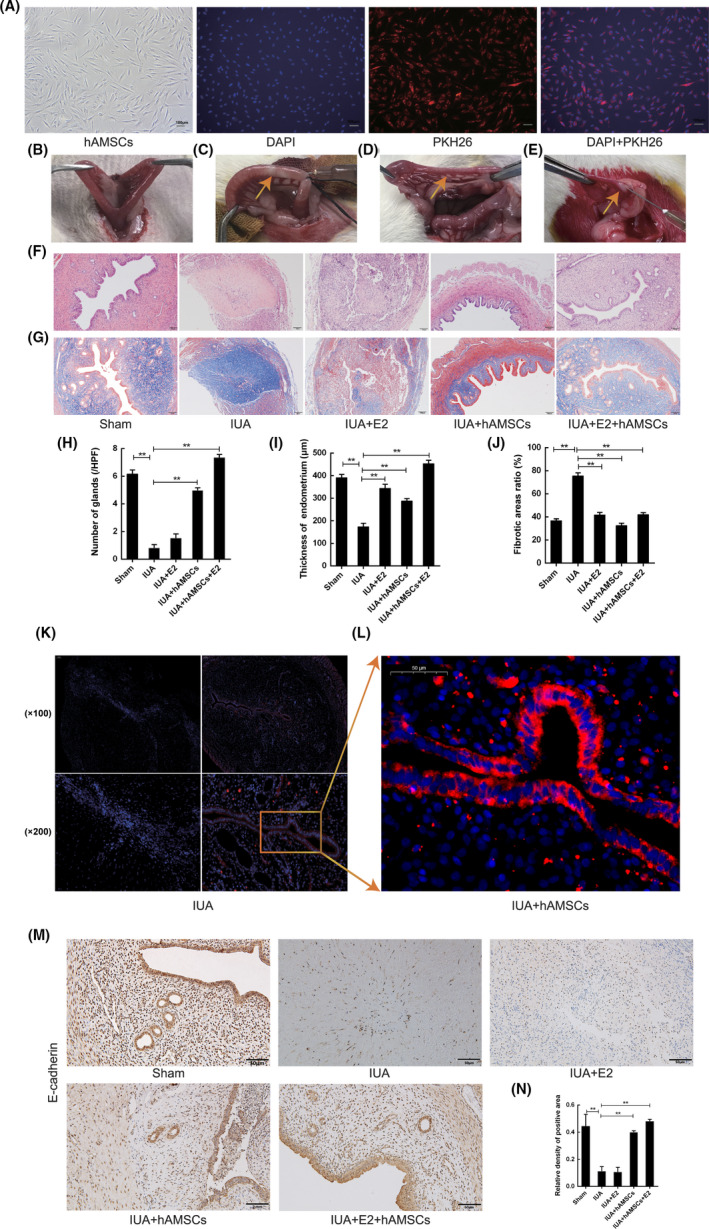
Endometrial regeneration and repair by hAMSCs transplantation. (A) PKH‐marked hAMSCs grew healthily, with cell membrane being red (PKH26) and the nucleus being blue (DAPI). (B) The diameter of the right uterus was in line with the left uterus of rats in the sham operation group. (C) The right uterus turned into white after being burnt with 95% alcohol for 3 min. (D) The right uterus where the modelling surgery was performed became harder and narrower 2 weeks after modelling. (E) No cell suspension flew out from the fimbriae and vaginal orifice of rats which were implanted with marked hAMSCs. (F, H, I) The number of endometrial glands and analysis of endometrial thickness of rats in each group after H&E staining. **p < 0.05. (G, J) Analysis of the percentage of fibrosis area in each group after Masson staining. **p < 0.05. (K) In vivo tracking of hAMSCs marked with PKH26. Immunofluorescence result of PKH26‐marked hAMSCs in the uterus two weeks after transplantation. (L) Enlargement of rectangular box in K. (M) Recovery of epithelial cells in the uterus of each group detected with Immunohistochemistry two weeks after PKH26‐marked hAMSCs transplantation. (N) Statistical analysis of the percentage of positive E‐cadherin area. **p < 0.05

PKH26‐labelled hAMSCs grew healthily with intact cell membranes and nuclei and were adherent. No suspended cells (Figure 2A) were observed. The uterus of normal SD rats is shaped like ‘Y’ (Figure 2B). The right uteruses of the rats turned white after injury with 95% alcohol for 3 min (Figure 2C). The site became hard. Following the generation of the IUA model, the uterine cavity became harder and thinner after 2 weeks (Figure 2D).

H&E staining demonstrated that the uterine cavity in sham‐operated animals was morphologically normal, with endometrial glands throughout the submucosal tissue and the basal layer, and columnar epithelial cells covering the endometrium. Compared with sham‐operated animals, the IUA group showed considerably reduced uterus volume, with densely adhered uterine walls, significantly reduced number of endometrial glands (1.14 ± 0.37), no columnar epithelial cells on the endometrium and significant thinning of the endometrium. All these changes indicated successful modelling. Compared with the IUA group, the therapeutic effect in the IUA + E2 group was not significant. The endometrium showed thickening, but the morphology of the uterine cavity did not improve, and the number of endometrial glands (1.69 ± 0.57) and the growth of endometrial epithelial cells did not increase. Compared with the IUA group, the IUA + hAMSCs group showed restored morphology of the uterine cavity, with columnar epithelial cells covering the endometrial surface and increased endometrial thickness as well as increased number of glands (4.96 ± 0.80). However, the therapeutic effect in the IUA + hAMSCs + E2 group was the best, with morphological restoration of the uterine cavity, significant growth of a single layer of columnar epithelial cells, significant thickening of the endometrium and considerable increase in the number of glands (6.77 ± 0.78) (Figure 2F). The IUA + hAMSCs and IUA + hAMSCs + E2 groups presented remarkably increased number of endometrial glands compared with the IUA group (*p* < 0.05). Endometrial thickening starkly differed in the IUA + E2, IUA + hAMSCs and IUA + hAMSCs + E2 groups compared with the IUA group (*p* < 0.05) (Figure 2H,I).

Masson staining revealed an even distribution of collagen fibres in the sham animals, but showed considerably darker signals in the IUA group (*p* < 0.05). Endometrial fibrosis was remarkably alleviated in the IUA + E2, IUA + hAMSCs, and IUA + hAMSCs + E2 groups compared with the IUA group (*p* < 0.05) (Figure 2G,J).

Under a fluorescence microscope, it was observed that PKH26‐labelled hAMSCs were mainly distributed in the endometrial epithelium 2 weeks post‐transplantation. No red fluorescence was observed in the rat uterus in the IUA group (Figure 2K,L).

Immunohistochemistry revealed that E‐cadherin expression was not significant in the IUA and IUA + E2 groups, but was markedly elevated in endometrial epithelial cells in the IUA + hAMSCs and IUA + hAMSCs + E2 groups (*p* < 0.05) (Figure 2M,N), suggesting differentiation into endometrial epithelial cells.

### 3.3 Notch pathway's function in the treatment of IUAs by hAMSC transplantation

**FIGURE 3:**
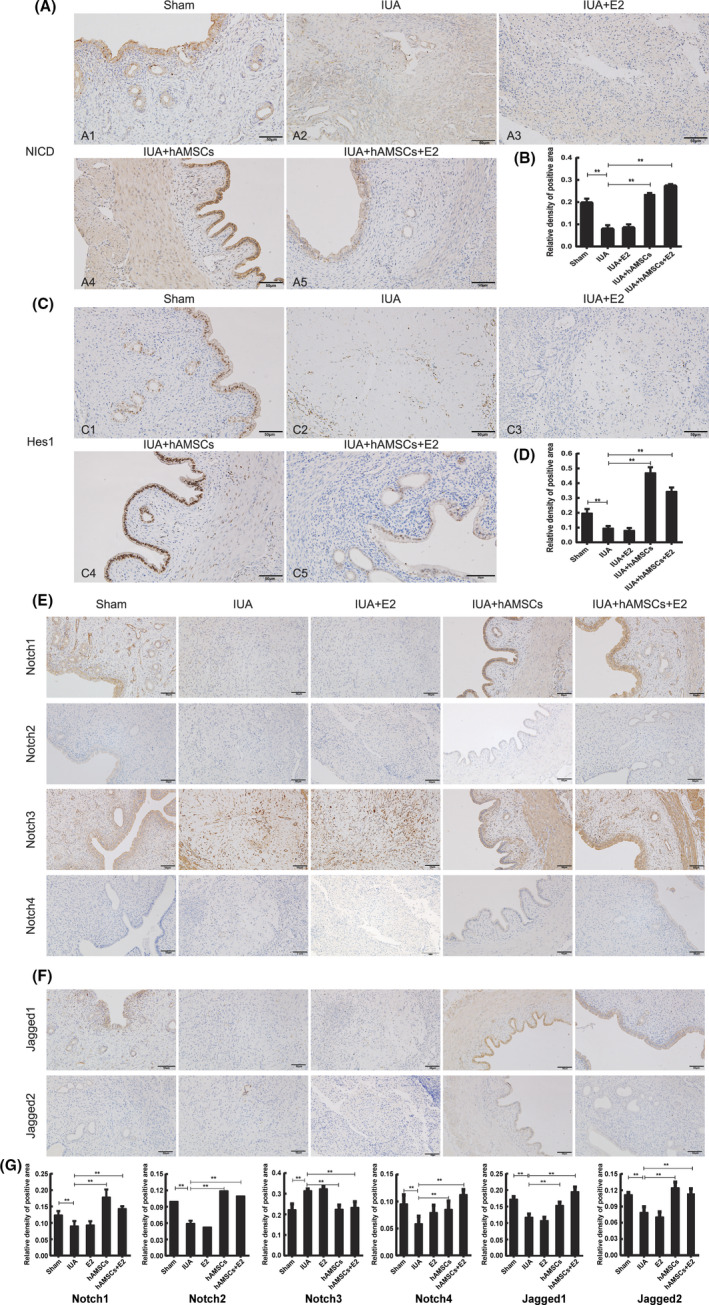
Role of Notch signalling pathway in the treatment of IUA with hAMSCs transplantation. (A) Immunohistochemical analysis of NICD expression in each group. (C) Immunohistochemical analysis of Hes1 expression in each group. (E) Immunohistochemical analysis of Notch receptor (Notch1‐4) expressions in each group. (F) Immunohistochemical analysis of Notch ligand (Jagged1‐2) expressions in each group. (B, D, G) Three high‐power fields (ie 40×) were randomly selected for each section for analysis. Image‐Pro Plus (Version 6.0) Image analysis software was used to estimate the percentage of the positive staining area, and then the average value was taken as the relative expression level of each protein. The results showed that activation of Notch signalling pathway benefits the treatment of IUA with hAMSCs transplantation. **p < 0.05

To explore the function of Notch signalling in hAMSC transplantation, we detected the expression levels of *NICD* and its downstream target gene *Hes1*. The *NICD* and *Hes1* levels were significantly decreased in the IUA and IUA + E2 groups (*p* < 0.05) compared with those in the sham animals and were increased in the IUA + hAMSCs and IUA + hAMSCs + E2 groups compared with those in the IUA group (*p* < 0.05) (Figure 3B,D). *NICD* and *Hes1* were mostly detected in the cytosol and the nucleus, respectively (Figure 3A,C). Furthermore, uterine levels of Notch receptors (Notch1–Notch4) and Notch ligands (Jagged1 and Jagged2) were assessed in each group by immunohistochemistry. Notch1, Notch3 and Jagged1 were detected on the cell membrane in all groups, as opposed to Notch2, Notch4 and Jagged2 (Figure 3E,F). Notch1 and Jagged1 expression was reduced in the IUA and IUA + E2 groups (*p* < 0.05) compared with that in the sham animals, but was elevated in the IUA + hAMSCs and IUA + hAMSCs + E2 groups compared with that in the IUA group (*p* < 0.05). In contrast, Notch3 expression in the uteruses was markedly elevated in the IUA and IUA + E2 groups compared with that in sham rats (*p* < 0.05) and decreased in the IUA + hAMSCs and IUA + hAMSCs + E2 groups compared with that in the IUA group (*p* < 0.05) (Figure 3G).

### 3.4 Differentiation of hAMSCs into endometrial epithelial cells in vitro

**FIGURE 4:**
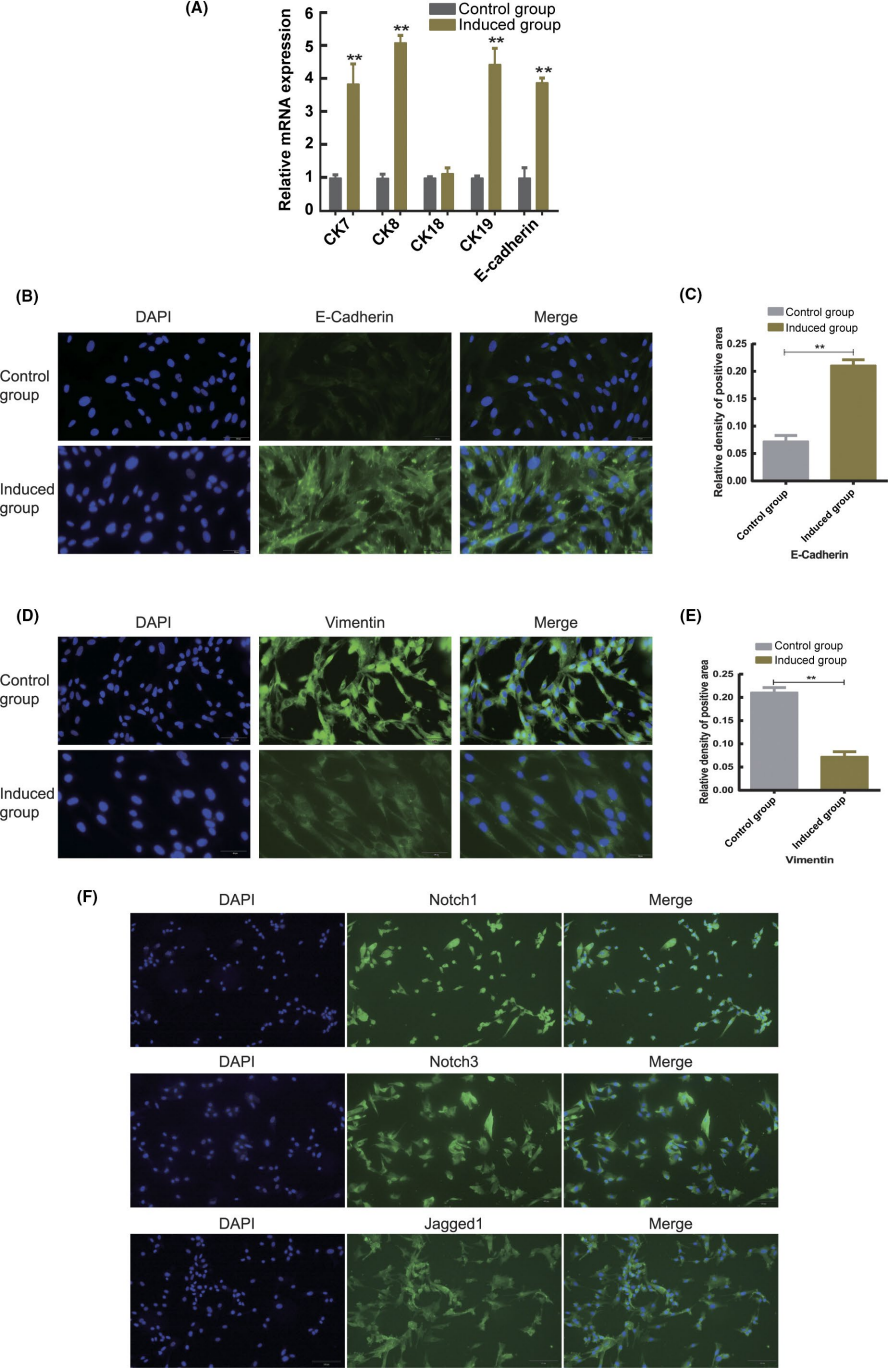
Differentiation of hAMSCs into endometrial epithelial cells in vitro. (A) Analysis of the expression of mRNA of epithelial markers (CK‐7, CK‐8, CK‐18, CK‐19 and E‐cadherin) before and after hAMSC differentiation. The expressions of mRNA of epithelial markers significantly increased after hAMSC transplantation, with significant difference as compared with the control group (*p* < 0.05). (B, D) E‐Cadherin and vimentin were detected by immunofluorescence, with target proteins fluorescing green and the nucleus fluorescing blue. E‐Cadherin and vimentin were observed in the cytoplasm. hAMSCs started to differentiate into endometrial epithelial cells 3 days after induction. (C, E) Analysis of the expression of E‐cadherin and vimentin before and after hAMSC differentiation. The results suggested that the expression of E‐Cadherin considerably increased but that of vimentin decreased, with significant difference as compared with the control group (*p* < 0.05). (F) Notch1, Notch3 and Jagged1 were detected by immunofluorescence, with target proteins fluorescing green and the nucleus fluorescing blue. Notch1, Notch3 and Jagged1 were observed in hAMSCs

To simulate the uterine environment, endometrium‐conditioned medium was used to promote hAMSC differentiation into target cells. The induction conditions included growth factors (TGF‐β1, EGF and PDGF‐BB) and 10^−6^ mol/L β‐estradiol + endometrium‐conditioned medium. Three days after culturing, RNA was extracted from hAMSCs in the control and induction groups to detect the mRNA levels of epithelial markers (*CK*‐*7*, *CK*‐*8*, *CK*‐*18*, *CK*‐*19* and *E*‐*cadherin*) by qRT‐PCR. Furthermore, E‐cadherin and vimentin expression was also detected by immunofluorescence. Compared with the control levels, the mRNA levels of *CK*‐*7*, *CK*‐*8*, *CK*‐*19* and *E*‐*cadherin* were considerably increased in the induction group (*p* < 0.05), as opposed to the *CK*‐*18* level, which was decreased (Figure 4A). Immunofluorescence (Figure 4B,D) indicated elevated expression of vimentin and reduced level of E‐cadherin in the control rats compared with those in the induction group (Figure 4C,E; *p* < 0.05).

Notch1, Notch3 and Jagged1 levels in hAMSCs were examined by immunofluorescence, and the results showed positive expression (Figure 4F).

### 3.5 Regulation of Notch signalling in hAMSC differentiation into endometrial epithelial cells

**FIGURE 5:**
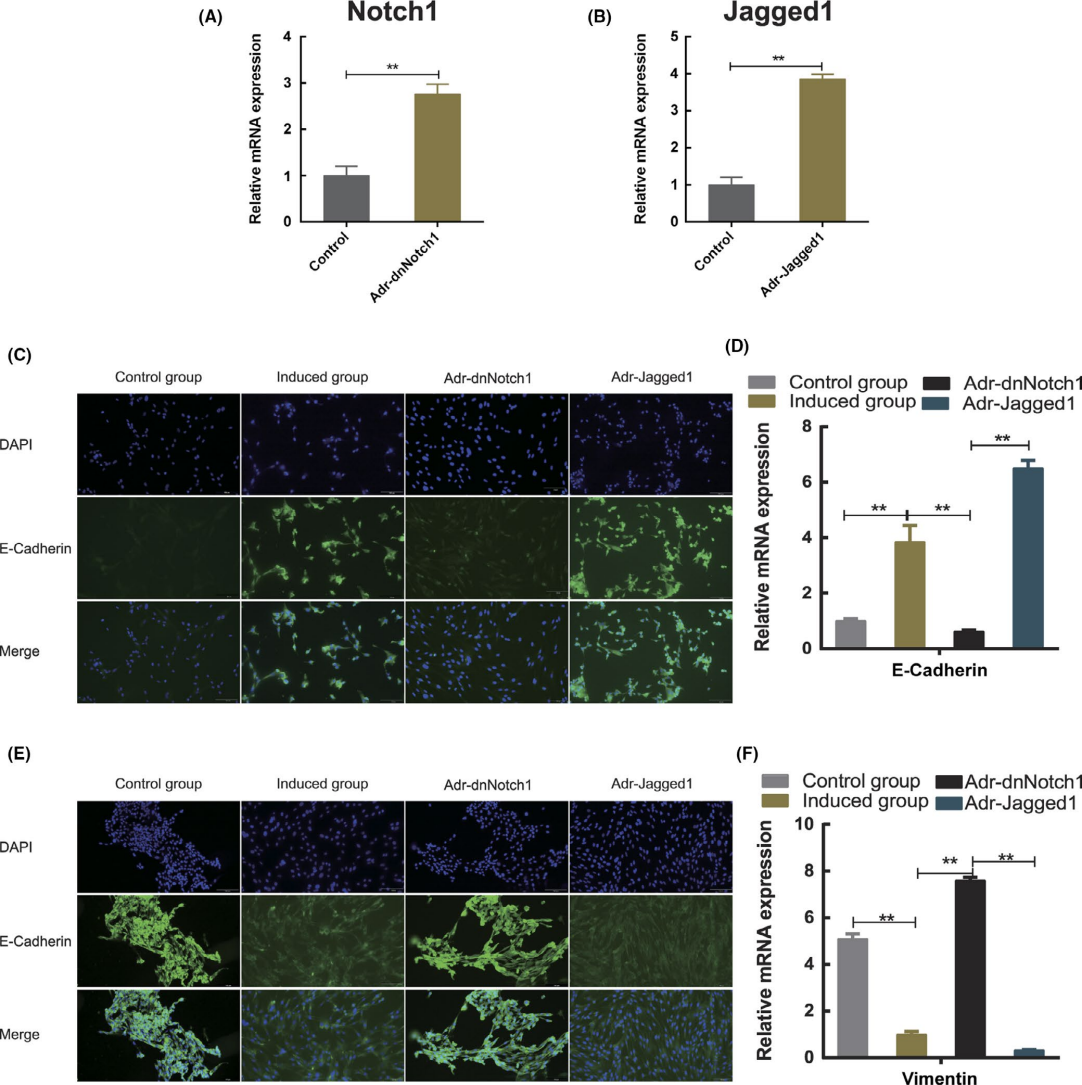
Effects of Notch signalling pathway intervention on hAMSC differentiation. (A) Adenovirus vector AdR‐dnNotch1 is capable of infecting hAMSCs. The expression of Notch1 mRNA in hAMSCs infected with AdR‐dnNotch1 increased markedly compared with that in hAMSCs infected with Ad‐RFP (*p* < 0.05). (B) Adenovirus vector AdR‐Jagged1 is capable of infecting hAMSCs. The expression of Jagged1 mRNA in hAMSCs infected with AdR‐Jagged1 significantly increased compared with that in hAMSCs infected with Ad‐RFP. The difference was significant compared with the control group (*p* < 0.05). (C, E) E‐Cadherin and vimentin were detected by immunofluorescence, with target proteins fluorescing green and the nucleus fluorescing blue. (D) Analysis of the expression of E‐cadherin in each group showed that the expression of E‐cadherin increased in the induction group compared with that in the control group, but decreased in the inhibition group compared with that in the induction group and also elevated in the activation group compared with that in the inhibition group, with a significant difference (*p* < 0.05). (F) Analysis of the expression of vimentin in each group showed that the expression of vimentin was reduced in the induction group compared with that in the control group, with a significant difference (*p* < 0.05), and that it increased in the inhibition group compared with that in the induction group, but decreased in the activation group compared with that in the inhibition group, with a significant difference (*p* < 0.05)

To confirm the role of Notch signalling in hAMSC differentiation into endometrial epithelial cells, Notch signalling was blocked by the binding of the adenoviral vector AdR‐dnNotch1 product to the Notch ligand (inhibition group). Transduction was confirmed by significant overexpression of *Notch1* mRNA (Figure 5A). The level of E‐cadherin was significantly reduced, whereas that of vimentin was considerably elevated in the inhibition group compared with those in the induction group (*p* < 0.05) (Figure 5D,F), suggesting that blocking Notch signalling significantly inhibited hAMSC differentiation into endometrial epithelial cells.

We also transfected hAMSCs with the adenoviral vector AdR‐Jagged1 (activation group) and confirmed the transduction by overexpression of *Jagged1* mRNA (Figure 5B). The *E*‐*cadherin* mRNA level in hAMSCs was considerably increased, whereas the vimentin level was reduced in the activation group compared with those in the inhibition group (*p* < 0.05) (Figure 5D,F). Overall, the above findings suggested that Notch signalling induction promoted hAMSC differentiation into endometrial epithelial cells.

### 3.6 Influence of Notch signalling on cell cycle distribution during hAMSC differentiation

**FIGURE 6:**
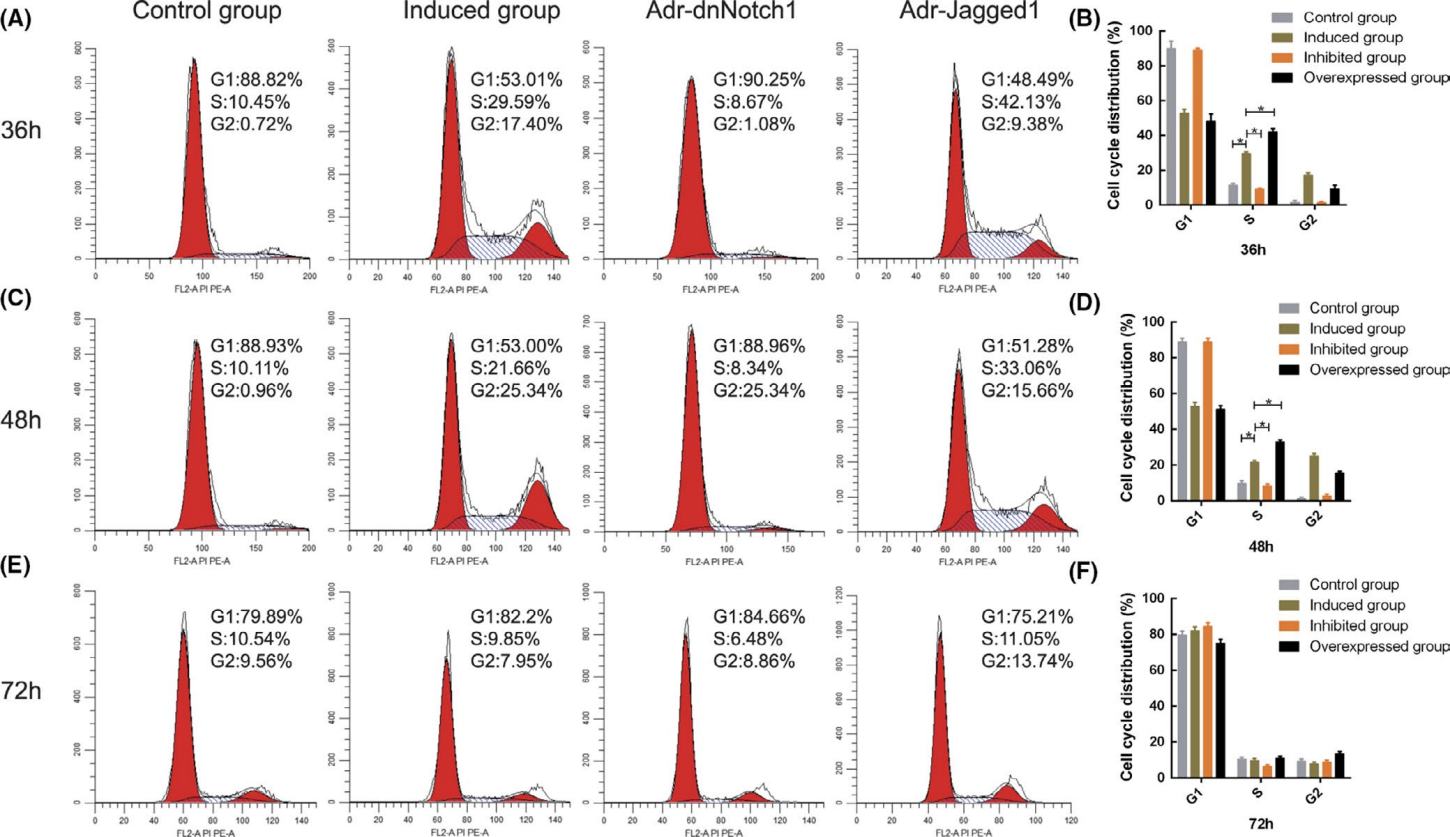
Flow cytometry of the distribution of cell cycles in each group. The distribution of cell cycles was analysed 36, 48 and 72 h after cell culture. (A–D) An increased number of cells in phase G1 transitioned rapidly into phase S in the induction group compared with that in the control group 36 and 48 h later. However, the number of cells in phase S decreased markedly with the inhibition of Notch signalling pathway. The number of cells in phase S elevated markedly in the activation group compared with that in the inhibition group (*p* < 0.05). (E, F) Analysis of cell cycles in each group suggested that there were no significant differences in the distribution of cells in different phase 72 h after cell culture, indicating early induction promoted the proliferation of cells

Flow cytometry revealed that the induced microenvironment at 36 and 48 h affected cell cycle distribution in hAMSCs and promoted the G1/S transition. The activation of Notch signalling accelerated the G1/S transition, whereas its inhibition caused the majority of cells to remain in the G1 phase, with a few progressing to the S phase (Figure 6A,C). These differences were significant (*p* < 0.05) (Figure 6B,D). There were no marked changes in cell cycle distribution among all groups at 72 h (Figure 6E,F).

## 4 DISCUSSION

Common treatment options for IUA encompass hysteroscopic adhesiolysis, hormone therapy and physical barriers. Despite great progress in surgery, recurrent IUAs after treatment remain a challenge.^28^ In our previous research, we found that the recurrence of treated IUAs could be effectively prevented by hAMSC transplantation.^18^, ^29^ Here, we studied the functions and mechanisms of Notch signalling in IUA treatment with hAMSC transplantation; Notch pathway's function in the treatment of uterine adhesions by hAMSCs was partially confirmed by in vivo experiments in SD rats. The results showed that hAMSC transplantation combined with oestrogen treatment was more effective than either of them alone in restoring uterine cavity morphology and promoting endometrial recovery. We also demonstrated that hAMSCs undergo adult endometrial epithelial cell differentiation after transplantation into the uterine cavity. Furthermore, the expression of Notch pathway proteins (NICD, Hes1, Notch1 and Jagged1) was significantly higher with the hAMSC treatment and the combined oestrogen treatment, which highlights Notch signalling as a key mediator of hAMSC differentiation to endometrial epithelial cells.

The growth factors TGF‐β1, EGF and PDGF‐BB, along with oestrogen and endometrial‐conditioned medium, induce the differentiation of bone marrow MSCs into endometrial epithelial cells in cell cultures.^27^, ^30^ In this study, we confirmed that an induction microenvironment composed of the above components was sufficient for hAMSC differentiation into endometrial epithelial cells, as indicated by significantly upregulated epithelial markers and downregulated mesenchymal markers. Furthermore, interfering with the Notch signalling pathway (inhibition and activation), using the Notch1‐dominant‐negative mutant adenovirus (Adr‐dnNotch1) and Jagged1‐overexpressing adenovirus (Adr‐Jagged1), affected the differentiation of hAMSCs in the induction microenvironment. Inhibition of Notch signalling suppressed hAMSC differentiation, whereas the activation of Notch signalling promoted hAMSC differentiation into endometrial epithelial cells.

In this study, we examined the Notch pathway's role in the treatment of IUAs by hAMSC transplantation, but whether hAMSCs differentiate into endometrial stem cells and whether their role in rats would change after interfering with Notch signalling remain to be explored.

In conclusion, the present study confirmed that the activation of Notch signalling promotes hAMSC differentiation into endometrial epithelial cells, which in turn promotes regenerative repair of the endometrium in uterine adhesions. In the future, the therapeutic benefits of Notch pathway intervention before hAMSC transplantation for endometrial injuries should be explored.

## CONFLICTS OF INTEREST

The authors have no potential conflict of interest to declare.

## AUTHOR CONTRIBUTION

**Jie Yu:** Conceptualization (equal); Data curation (equal); Formal analysis (equal); Investigation (equal); Methodology (equal); Project administration (equal); Resources (equal); Software (equal); Validation (equal); Writing‐original draft (equal); Writing‐review & editing (equal). **Wen wen Zhang:** Conceptualization (equal); Data curation (equal); Formal analysis (equal); Funding acquisition (equal); Methodology (equal); Project administration (equal); Resources (equal); Writing‐review & editing (equal). **Yue Jia Huang:** Validation (equal). **Ting Ya Gou:** Software (equal). **Cong Cong Sun:** Visualization (equal). **Feng Ying Zhang:** Formal analysis (equal). **Hua Yan Mao:** Software (equal); Validation (equal). **Yuan Ben Wu:** Investigation (equal). **Jiang Chang Li:** Software (equal). **Zhou Ni Liu:** Validation (equal). **Ting Ting Wang:** Software (equal). **Ren Ji Huang:** Software (equal). **Jia Wang:** Conceptualization (equal); Data curation (equal); Formal analysis (equal); Funding acquisition (equal); Methodology (equal); Project administration (equal); Supervision (equal).

## Data Availability

The data generated and/or analysed in the current study are included in this manuscript.
